# Exploring Usability and Patient Attitude towards a Smart Hospital Service with the Technology Acceptance Model

**DOI:** 10.3390/ijerph19106059

**Published:** 2022-05-16

**Authors:** Jui-Che Tu, Shi Chen Luo, Yi-Lin Lee, Ming-Feng Shih, Shu-Ping Chiu

**Affiliations:** 1 Graduate School of Design, National Yunlin University of Science & Technology, Yunlin 640301, Taiwan; tujc@yuntech.edu.tw; 2 Bachelor Program in Industrial Technology, National Yunlin University of Science & Technology, Yunlin 640301, Taiwan; cacaca710@gmail.com; 3 National Taiwan University Hospital Yunlin Branch, Yunlin 640301, Taiwan; 4 Lin Fengmian Academy of Fine Arts, Jiaying University, Meizhou 514015, China; ping_ya2001@yahoo.com.tw

**Keywords:** smart service system, use willingness and attitude, technology acceptance model

## Abstract

The demand for health care has increased with the development of global technology and the rise of public health awareness, and smart service systems have also been introduced to medical care to relieve the pressure on hospital staff. However, the survey found that patients’ willingness to use smart services at the time of consultation has not improved. The main research purpose of this study was to understand the willingness of patients from various groups to use smart medical service systems and to explore the influencing factors on patients’ use of smart service systems in hospitals through the technology acceptance model. This study distributed questionnaires in the outpatient area of National Taiwan University Hospital Yunlin Branch, and a total of 202 valid questionnaires were obtained. After related research and regression analysis, it was found that patients paid more attention to the benefits and convenience brought by smart services. If patients believed that smart services were trustworthy and beneficial to themselves, their usage intention and attitude would be positive. The results of this study are summarized by the following four points: (1) Designed according to the cultural conditions of different regions; (2) think about design from the patient’s perspective; (3) strengthen the explanation and promotion of smart services; and (4) add humanized care and design. This study could be used as a reference for hospitals to improve their service quality and systems in the future.

## 1. Introduction

With the development of the economy and technology as well as improvement in health awareness, the average life expectancy worldwide is on the rise [1], people are no longer satisfied with meeting a basic quality of life, and they are increasingly concerned about medical care, mental health, and service quality [2]. The demographic structure of the population is changing in line with rising medical standards and is becoming ageing. The global population is expected to reach 9.7 billion by 2050 [3], but families still suffer from a declining fertility rate, putting pressure on the overall productivity of society and the medical system. In comparison to other Asian countries, Taiwan’s elderly population rate is the second highest, and the aging of the population is serious in all counties and cities. Chiayi County has the highest rate (18.61%) within all the counties, followed by Yunlin County with 17.69% [4]. With the phenomenon of childlessness in society, the traditional family division of labor has been changed, and it is estimated that the dependency ratio in Taiwan will be raised to 67.3% in 2050, which means that every 1.5 young adults will be responsible for one elderly person [5]. This will lead to an increasing in dependency problems and a growing demand for government or social health care. According to the statistics of the Ministry of Health and Welfare (Taiwan) in 2019, the average number of hospital visits by Taiwanese people per year is 17, the departments with the highest number of outpatient visits are family medicine and internal medicine, and the number of people visiting a hospital continues to rise, as found from the statistics on the number of medical visits from 2017 to 2019 (Table 1).

Due to the problems of medical services and human negligence that follow an aging society, it has become necessary to optimize the outpatient caring procedure. Therefore, the use of technology to reduce the pressure on insufficient medical resources has been one of the world’s concerns in recent years. According to the Healthcare Information and Management Systems Society (HIMSS) global survey, the United States is a global leader in the development of smart hospitals, with 1991 hospitals certified to HIMSS EMRAM Level 7 [7]. EMRAM classifies healthcare organizations into eight levels of information technology, from 0 to 7, with the highest level, Level 7, being paperless and able to fully implement information management in hospital management, clinical decision-making, and quality of care. Although the Asia-Pacific region has been slower to get started than developed countries such as the United States, it is actively catching up. China is actively promoting smart healthcare, building smart hospitals and constructing a new state of smart healthcare development to improve the modern management of healthcare and meet the growing demand for healthcare from the people [8]; Japan plans to establish ten demonstration hospitals implementing “AI medicine” by 2022, using AI technology to automatically record cases, tests, and diagnostic protocols [9]; Taiwan’s Lin Kou Chang Gung Hospital and China Medical University Hospital received EMRAM Level 7 accreditation in June 2019 and December 2019 [10,11].

The World report on ageing and health has been published by the World Health Organization in 2015, and suggested that a “people-centered” approach should be adopted to improve access to medical care services, so that all people can receive safe, immediate, and efficient healthcare services that meet their preferences and needs [12]. Therefore, with the popularization of smartphones and the development of internet convenience, medical services are moving toward the direction of being smarter and more efficient, which has transformed the traditional medical service methods, obtaining more efficient services through the assistance of smart technologies. Self-service technologies (SSTs) can enable users to use services without contacting service personnel and allow users to assume the role of traditional service personnel. SSTs have three major service functions: customer services, transactions, and self-service [13]. With the development of society and technology, SSTs have produced great changes in the service effect between users and enterprises, as they have not only given users more control over the service process but also reduced the workload of service providers [14]. The traditional medical service structure is encountering the problems of insufficient manpower and the unequal distribution of resources. In the face of the reduction of the labor force in an aging society, medical self-services can undoubtedly reduce the pressure on medical personnel, optimize the patient’s medical experience, and improve hospital operational efficiency. Hsieh [4] proposed four main types of self-service, namely, telephone & interactive voice response systems, internet, interactive kiosks, and video/CD technologies. Compared with the existing smart medical services in the hospital concerned, the applications of SSTs are described in Table 2.

With the rapid development and advancement of technologies, technology-based self-service is also constantly developing. However, if the self-service technology is too complex, customers may not be able to adapt to the changing technological progress, leading to customers’ avoidance in using these services [16]. How to achieve a friendly connection between the interaction of humans and machines will affect users’ perceptions of and attitudes toward the service system [17]. According to the Annual Outpatient Experience Survey Report 2020 of Taipei Veterans General Hospital, regarding its mobile service app, which has been vigorously promoted and publicized in recent years, only 6.9% of the respondents used it for registration and 0.8% used it for making payments, and the penetration rate was obviously insufficient [18]. People’s attitudes can be used to predict behavioral intentions [19], and attitudes toward a particular behavior may have a decisive effect on the strength of a person’s intention to perform that behavior, which in turn may affect the person’s actual performance of that behavior [20,21]; therefore, people’s perception of and attitude toward services received during an outpatient visit may also affect their behavior intention. The use of a good service system and smart technology configuration can support the hospital management system in being more sophisticated and completing in implementation, and also make it easier for patients and medical-related users to operate and understand the system. As found by related research, medical service experience has a significant impact on both the quality of the patient relationship and the loyalty of the patients [22,23]. Therefore, for hospitals, improving the patient’s medical experience will help the hospital to establish a good reputation and image. As this increases the percentage of patients who are willing to come to the hospital again, it is also a key factor affecting the long-term success of medical institutions [24].

This study adopted both quantitative and qualitative methods, using semi-structured interviews and questionnaires as the main research methods. Through interviews with three experts in the academic and industrial fields, we will learn about the current status and development trend of smart hospital services, and discuss with them the factors which enhance patients’ willingness to use them. The interviews was conducted from 4 March 2021 to 30 March 2020.The questionnaire survey was designed to understand outpatients’ willingness and attitude toward the use of hospital smart services, using the TAM as the main framework and adjusting it with the results of expert interviews. This phase of the questionnaire survey was conducted one-on-one with patients in the outpatient clinic of the hospital, and the researcher would assist the patients in completing the questionnaire, in order to make the respondents fully understand the research direction of the study and enhance the validity of the questionnaire. The statistical analysis methods used for questionnaire analysis were descriptive statistics, correlation analysis, regression analysis, *t*-test, and one-way ANOVA.

With the cooperation of National Taiwan University Hospital Yunlin Branch, the outpatient area of this hospital was selected as the research field of this study. Through targeting the walk-in registration method for outpatient services, self-service kiosks for chronic disease patients to refill prescriptions, self-check-in kiosks, and self-payment kiosks, this study focused on analyzing patients’ willingness to use the smart service system when seeking medical treatment in the outpatient clinic. It was expected that, through collecting patients’ opinions in this research, the hospital could discover the pain points and needs of its medical consultation service system, and the patient’s willingness to use the hospital smart service system could be improved and enhanced. Furthermore, the advantages of the hospital smart service system could also be enhanced. The main purposes of this study can be summarized into four points:

1. To understand the development status and development trend of the hospital smart service system.
2. To analyze patients’ willingness to use and accept such services through a technology acceptance model.
3. To explore the influencing factors on patients’ use of the hospital smart service system.
4. To further develop the design strategy of the future hospital outpatient smart service system and promote the expansion and the advantages of smart medical applications.

## 2. Materials and Methods

### 2.1. Research Structure

**Figure 1:**
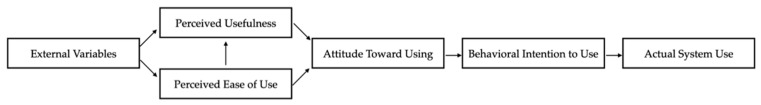
Technology acceptance model [27].

In order to study how individuals’ attitudes affect their behavior consciously, Ajzen and Fishbein [25] proposed the theory of reasoned action (TRA). Although this theory has been widely used by experts and scholars in many fields to explore the relationship among individuals’ attitudes, subjective norms, and behavioral intentions, it is still unable to effectively explain the behavior of individuals in the field of information technology [26]. Therefore, the technology acceptance model (TAM) (as shown in Figure 1) was proposed based on TRA to explore the influence of external variables on individuals’ beliefs, attitudes, and intentions. By understanding the degree of users’ usage intentions in using information technology and the degree of influencing willingness, the model can predict whether behavioral intention affects an individual’s information technology use behavior [27,28,29]. Many researches have used the TAM model to explore users of health information systems [30,31]. Some research added externalities (gender, age, experience, etc.) as adjustment factors and thus were able to predict more than 70% of users’ intentions [32].

Davis and Davis [27] pointed out that the two aspects of an individual’s perceived usefulness and perceived ease of use are the main determinants of accepting and using new information technology, and that when a system is considered as easier to use by users, it is usually considered more useful too. Perceived usefulness is usually defined as individuals’ subjective perception that the use of a particular information technology is beneficial to their work or efficiency [29]. In this study, perceived usefulness was defined as the degree to which patients believe that using the smart service system of the hospital is helpful for their own medical treatment, and perceived ease of use was defined as the user’s perceived ease of operation when using specific information technology [33]. In this study, a high perceived ease of use meant that the smart service system was easier for the patients to learn and use. In previous studies, the acceptance of new technologies by users usually depended on its potential to solve a main problem, without putting an excessive burden on the users [34]. In a medical space, both software (the interaction between doctors and patients) and hardware (service equipment and space design) will convey, replicate, and expose the values of the medical system to the patients [35]. Most patients’ attitudes toward medical quality are defined by their experience [36,37]. Relevant studies have also pointed out that medical service experience has a significant impact on the quality of doctor-patient relationships and patient loyalty [22,23]. Therefore, for hospitals, improving the patient’s medical experience will help the hospital to establish a good reputation and image, increase the proportion of patients who will consider choosing the hospital again [24], and affect the long-term success of medical institutions.

**Figure 2:**
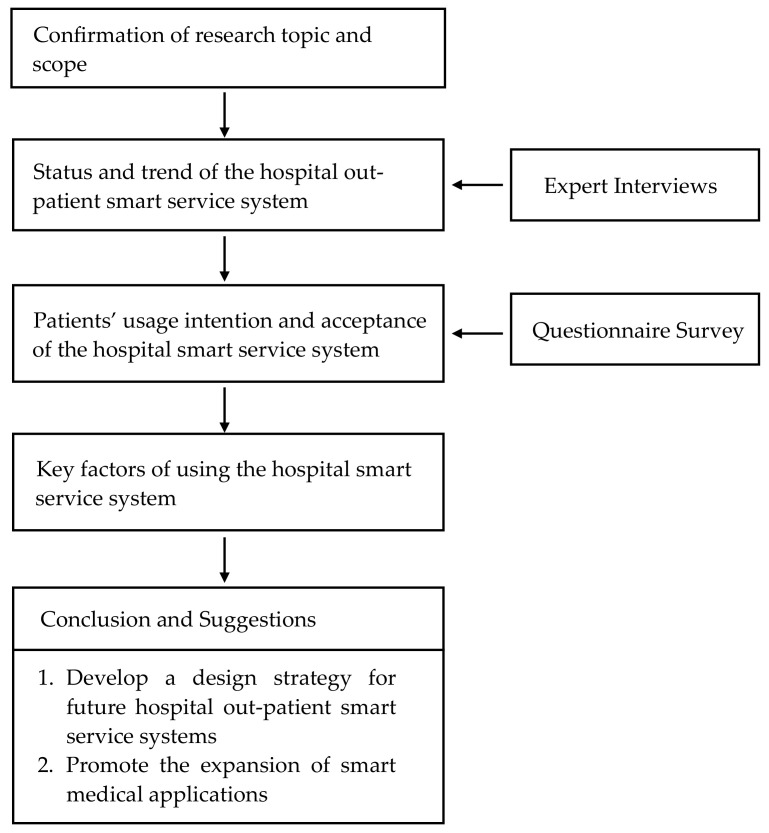
Research structure.

Therefore, this study intended to use the technology acceptance model (TAM), with a questionnaire survey and expert interviews as the main research methods, to analyze patients’ perceptions of and attitudes toward the hospital smart service system, as well as explore patients’ usage intention and acceptance, in order to facilitate the subsequent identification of the influencing factors of patients’ use of smart service systems for medical treatment. Based on the research topic and purpose, this study outlines its research structure and steps. First, the research direction was proposed according to the research topic. Secondly, the patients’ attitudes and willingness to use the hospital smart service system were analyzed using TAM. Finally, the research scope and main research subjects were defined, and the design strategy of the hospital smart service system was discussed through expert interviews and questionnaire surveys. The research was carried out in three stages, as shown in Figure 2.

### 2.2. Research Subjects

National Taiwan University Hospital (NTUH) Yunlin Branch was selected as the research subject of this study. NTUH Yunlin Branch’s Douliu Campus, located in Yunlin County, was established in 2004 and has rapidly improved the medical level of the Yunlin area. The purpose of NTUH Yunlin Branch is to promote people’s health awareness and provide medical services needed by the public. At the same time, it also actively promotes international medical services, establishes international medical service cooperation with other countries, and is committed to the exchange and management of international medical care [38]. With the goal of being a trusted hospital, NTUH Yunlin Branch continues to develop and innovate, and it has gradually become a model of telemedicine in Taiwan. It has successively promoted telemedicine services, such as remote care for wounds, and home isolation and quarantine communication diagnoses and treatments, while establishing patient-centered medical services [39]. In the hospital, smart technologies have also been introduced into the medical service process, including online registration services, self-payment kiosks, self-service prescription refill kiosks, and other service methods, so that people can obtain high-quality services when they see a doctor.

**Figure 3:**
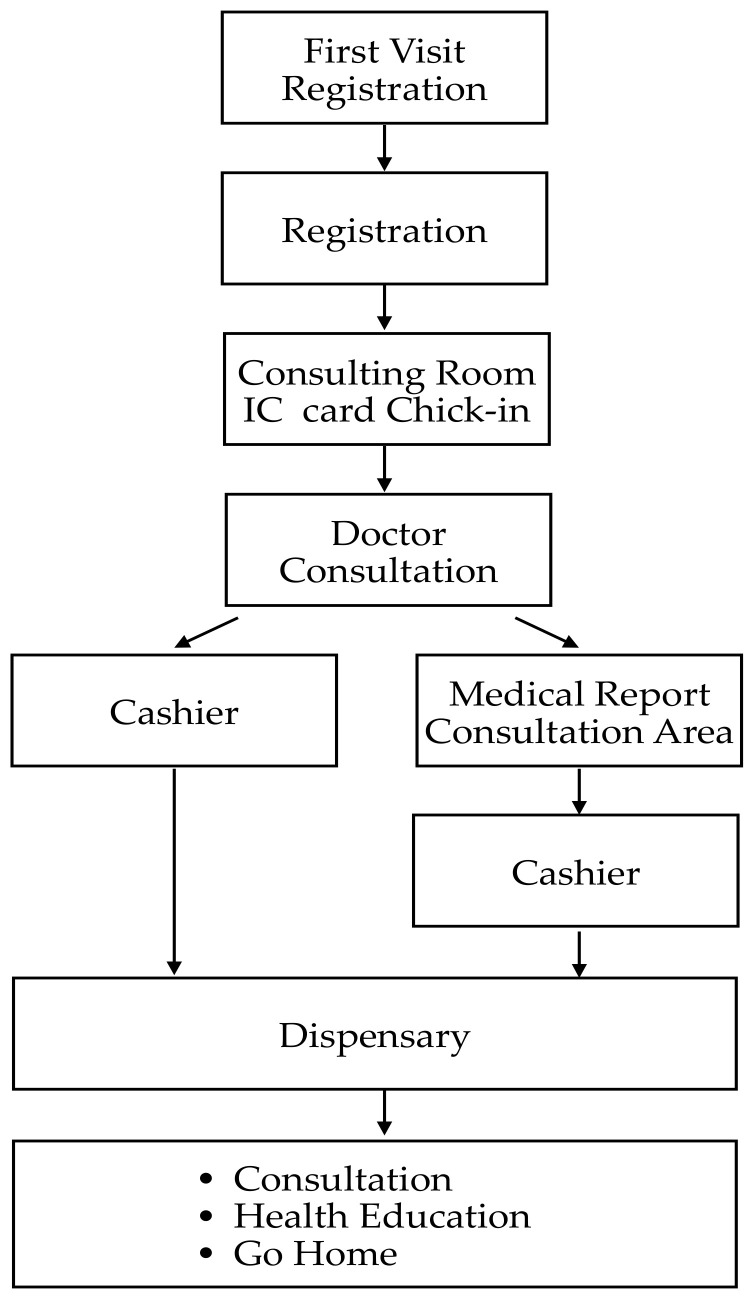
Out-patient care procedure.

In the outpatient process, the first step is to make an appointment. The patient can choose an online appointment, a telephone appointment, or an on-site appointment. Then, the patient checks in at the clinic by inserting the NHI card and waits in the waiting area for their appointment. When it is their turn, the patient will be called by voice with their name being shown on the display near the consulting room. After the diagnosis and treatment by the doctor, if there is no need for any further examination, the doctor will ask the patient to go directly to the cashier counter to pay the fees. If a follow-up examination is required, the fees will be paid after the examination. The patient can freely choose to pay at the counter or use the self-payment kiosk to pay the fees. Finally, the patient can go to the pharmacy to get the medicine or participate in consultation and health education courses. The specific flow chart is shown in Figure 3.

**Figure 4:**
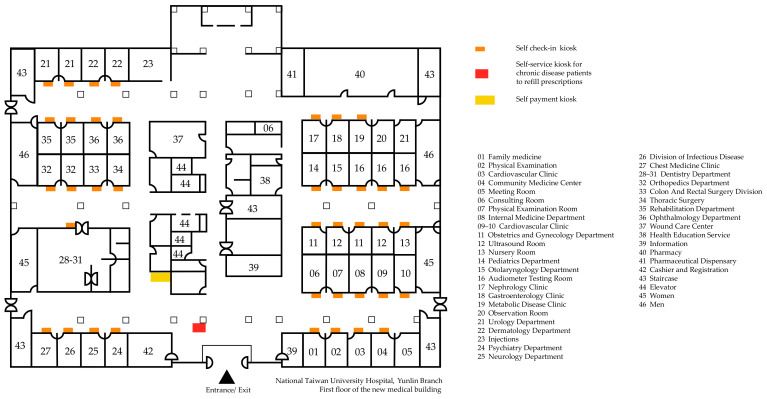
Hospital outpatient clinic floor plan.

In the spatial layout of NTUH Yunlin Branch, after a person enters from the gate, they will see the cashier and registration counter on the left. Two self-payment kiosks are located opposite the cashier and registration counter. There are also two self-service kiosks for refilling prescriptions near the exit of the hospital, and one self-check-in kiosk is located outside each department. The specific plan of the hospital is shown in Figure 4.

The most frequently used smart service in the outpatient process of the NTUH Yunlin Branch has been the appointment and registration, mobile service APP, self check-in kiosk, self payment kiosk and self-service kiosk for chronic disease patients to refill prescriptions. Details of each service are shown in Table 3.

### 2.3. Research Method

**Figure 5:**
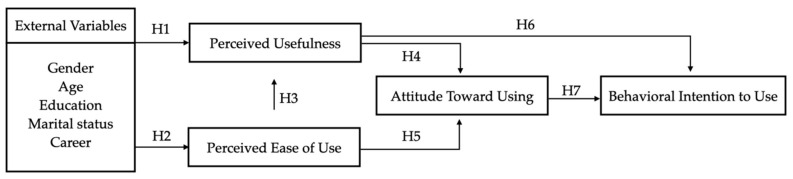
Structure of the research hypotheses.

Davis and Davis [27] suggested that users’ use of IT is influenced by two main factors, namely perceived usefulness and perceived ease of use, and proposed a TAM model based on the causal relationship between these two self-cognitive dimensions and actual actions. Since users’ cognition may be influenced by external factors, external influences need to be taken into account when exploring users’ behavioral attitudes [27,40], and in this study, external variables are defined as the demographic variables of the respondents. In previous studies, it has been confirmed that perceived ease of use affects perceived usefulness to some extent [41,42], and perceived ease of use and perceived usefulness are considered to be the main factors that directly or indirectly influence attitudes and behaviors [40,43]. In TAM, the person’s intention to use is determined by attitude and self-perception, which in turn is determined by self-perception, which in turn is influenced by different external factors [44]. Based on the literature review and related research, using the technology acceptance model as the research basis, the external variables, perceived usefulness, perceived ease of use, attitude, and behavioral intention were proposed in this study in combination with the demographic variables to explore the possible relationship among the variables and then deduce the research hypotheses of this study. The structure is shown in Figure 5.

The hypotheses of this study were as follows:

**H1.**  *External variables of patients’ perception of hospital smart service systems will positively affect perceived usefulness*.

**H2.**  *External variables of patients’ perception of hospital smart service systems will positively affect perceived ease of use*.

**H3.**  *Patients’ perceived ease of use of hospital smart service systems will positively affect perceived usefulness*.

**H4.**  *Patients’ perceived usefulness of hospital smart service systems will positively affect attitude*.

**H5.**  *Patients’ perceived ease of use of hospital smart service systems will positively affect attitude*.

**H6.**  *Patients’ perceived usefulness of hospital smart service systems will positively affect behavioral intention*.

**H7.**  *Patients’ attitudes toward using hospital smart service systems will positively affect behavioral intention*.

The purpose of this study was to explore the patients’ perception, experience, and usage behavior of the hospital smart service system. Therefore, it was necessary to first understand the current status of and trends in the hospital smart service system through expert interviews. In the second stage, patients who had visited NTUH Yunlin Branch’s outpatient clinic were taken as the research subjects. A questionnaire survey was used to further learn about the users’ perception of and attitude toward the smart service system, and the influence of various factors on the smart service system was analyzed. The research on the subjects was divided into two parts. The first part was an expert interview. The subjects included an expert from the medical smart equipment industry, an expert in caring and wellbeing design, and a medical worker. Semi-structured interviews were conducted with the three experts. The interview time was about 40 min to one hour. The backgrounds of the interview subjects are shown in Table 4.

In the second stage of the questionnaire survey, patients who had visited the NTUH Yunlin Branch’s outpatient clinic were the subjects. During weekdays, questionnaires were distributed to and random interviews were conducted with willing patients in the cashier and registration area, the waiting area, and the dispensary area of the outpatient clinic. The main questions of the questionnaire survey and interview included: (1) What is the patient’s attitude toward the hospital smart service? (2) What is the patient’s willingness to use the hospital smart service? What are the influencing factors? (3) What are the differences in the willingness to use hospital smart services due to demographic variables such as different ages or genders? The initial structure of the questionnaire was designed using the technology acceptance model as the main structure, and it was then adjusted based on the literature review. Scoring was based on a Likert five-point scale. The obtained information was statistically analyzed and sorted, and research conclusions and suggestions were made. The items in the questionnaire are shown in Table 5.

### 2.4. Data Analysis Methodology

Based on the above, the following analysis methodology will be used in this study.

1. Descriptive Statistics

Descriptive statistical analyze the basic data of the respondents to understand the actual situation of the sample.

2. *t*-test and one-way ANOVA

Since the external variables of the technology acceptance model were defined as the demographic variables of the respondents, independent sample *t*-test and one-way analysis of variance (ANOVA) were used to compare whether the external variables potentially affected the perceived usefulness and perceived ease of use of the respondents.

3. Analysis of Correlation

In order to explore the correlation of the effects among these dimensions, this study used analysis of correlation and regression analysis.

Analysis of correlation can be used to describe the degree of mutual correlation between two variables. Through Pearson’s correlation coefficient, the degree of correlation and closeness between the variables in each dimension can be analyzed and explained to understand the degree of correlation between different dimensions [45].

4. Regression Analysis

The purpose of regression analysis is to help researchers clarify the relationships among variables in various dimensions and then establish explanatory models and prediction models [46].

## 3. Results

Based on the research purpose and structure, this study used a literature review, expert interviews, and questionnaires to understand the current development of the hospital smart medical service system, explored the influencing factors of patients’ use of the hospital smart medical service system, and analyzed the factors influencing patients’ use of the hospital smart medical service system.

### 3.1. Summary of the Expert Interview Contents

During the interview process, audio recordings were mainly used as the recording method of this study, and the key points of the transcripts were copied to assist the recording without interfering with the ideas and directions of the interviewed experts. For the contents proposed by the three experts in the interview, NVivo software was adopted by this study to encode the verbatim transcripts, and the contents were summarized into five parts. The specific contents are shown in Table 6.

### 3.2. Questionnaire Analysis

#### 3.2.1. Reliability and Validity Analysis

A pre-test was performed before the formal investigation in this study to verify the reliability and validity of the questionnaire. There were 19 items in the questionnaire used for the pre-test, 33 copies of the questionnaire were issued, and 30 valid questionnaires were recovered. The results of the reliability analysis showed that the internal consistency coefficient of Cronbach’s Alpha was 0.983, indicating it had acceptable reliability and could be used as the formal questionnaire. A total of 202 questionnaires were collected for this survey. The Cronbach’s alpha coefficients for the variables of each dimension were all above 0.9, indicating that the consistency of the results was above the standard and that the results were reliable. The above dimensions and demographic variables were subjected to independent sample *t*-tests and analysis of variance to analyze the correlation between different physiological and psychological conditions of the patients and their acceptance of technologies, and regression analysis and correlation analysis were adopted to explore the influencing factors between various dimensions [22,26,27,47,48,49,50]. The questionnaire of this study was made by referring to and modifying the effective questionnaires of past scholars, and discussions were made with experts and scholars in related fields at the same time; therefore the questionnaire of this study had a correspondingly high validity.

#### 3.2.2. Analysis of the Respondents’ Basic Data

The demographic variables in this study included gender, age, educational level, marital status, occupational category, the internet devices previously used or currently using, and smart services used in medical treatment. There were seven items in total. Percentages of the collected data were calculated to explain the basic characteristics of the research statistics. Descriptive statistics are shown in Table 7.

Two multiple-choice questions had more than one answer in the questionnaire: the status of the respondents’ use of internet devices and the respondents’ use of hospital smart services. The survey found that more than 90% of the respondents had experience in using smartphones to access the internet and that more than 60% of them had experience in using computers. The respondents who had used tablets were relatively fewer than those who had used computers and smartphones. In terms of the proportion of using smart services, more than 80% of the respondents had used online registration services, and more than 60% of them had the experience of using self-payment kiosks and self-check-in kiosks, Fewer people had experience of using self-service kiosks for refilling prescriptions, with only 46 people.

#### 3.2.3. Analysis of the Respondents’ Technology Acceptance

Based on the proposed questionnaire and research structure, it was assumed by this study that the patient’s perceived ease of use of smart medical services would affect their perceived usefulness and then affect other dimensions. In order to explore the correlation of the effects among these dimensions, this study used correlation analysis and regression analysis. Correlation analysis can be used to describe the degree of mutual correlation between two variables. Through Pearson’s correlation coefficient, the degree of correlation and closeness between the variables in each dimension can be analyzed and explained to understand the degree of correlation between different dimensions [45]. The purpose of regression analysis is to help researchers clarify the relationships among variables in various dimensions and then establish explanatory models and prediction models [46].

The research dimensions in this study were divided into five dimensions: demographic variables, perceived usefulness, perceived ease of use, attitude, and behavioral intention. Because the external variables of the technology acceptance model were defined as the demographic variables of the respondents, whether the external variables would potentially affect the respondents’ perceived usefulness and perceived ease of use was tested through an independent samples *t*-test and one-way analysis of variance (ANOVA). The independent samples *t*-test analysis results are shown in Table 8 and Table 9. No significant difference was found between perceived usefulness and perceived ease of use among different genders, and there was no significant difference between the perceived usefulness of the males and the perceived usefulness of the females.

One-way independent sample ANOVA was used to analyze the correlation between the external variables and perceived usefulness, and the results are shown in Table 10 and Table 11. It was found that, for educational level, there were significant differences in perceived usefulness and perceived ease of use, and there was no significant difference in perceived usefulness and perceived ease of use for all other dimensions.

The Pearson correlation analysis revealed a positive correlation among perceived usefulness, perceived ease of use, attitude, and behavioral intention (Table 12). The results of the questionnaire survey were analyzed and verified by regression analysis (Table 13). It could be seen that each dimension had a significant relationship with its corresponding dimensions, which meant that when the patients’ perception of perceived usefulness, perceived ease of use, and attitude were better, they would be more likely to have a higher behavioral intention to use the hospital’s smart service systems.

**Figure 6:**
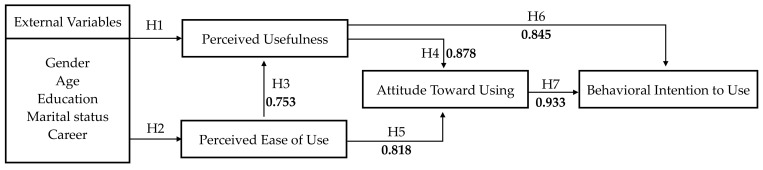
Research hypotheses results.

According to the aforementioned analysis results, the research hypotheses on the various dimensions constructed in this study, including the external variables, perceived usefulness, perceived ease of use, attitude, and behavioral intention, could be sorted out as shown in Figure 6. The patients’ perceptions of the hospital smart service system were summarized as shown in Figure 6. The patients’ attitude toward using the hospital smart system had the highest correlation with the impact on their behavioral intention, reaching 0.933, meaning that the patients would use the smart services if they had a positive attitude. The impact between the patients’ perceived usefulness and attitude was the second highest, reaching 0.878, indicating that whether the hospital smart services were beneficial to the patients’ consultation process was the key factor affecting their attitude. At the same time, the influence of perceived usefulness and the behavioral intention was 0.845, indicating that the patients believed the benefit of the smart services would also directly affect their behavioral intention to use the services. The conclusions shown in Table 14 were drawn based on the analysis results of the research hypotheses.

## 4. Discussion

According to the research data and the results of the data analysis, and echoing the purpose of this research, this study found three research conclusions. The detailed descriptions are listed below.

1. Social benefits of and users’ needs for hospital smart service systems

As could be seen through the literature review and the summary of the expert interviews, although the Asia-Pacific region started late in terms of smart services compared to developed countries in Europe and the United States, it has actively caught up in recent years [7,8,9,10,11]. Today, nearly all large hospitals in Taiwan offer smart medical services. In Taiwan, the proportion of smart service import in medical center hospitals is the highest for administrative services, with 43%; while in regional hospitals and district hospitals, the proportion of outpatient service import is the highest, with 26% and 14%, respectively [51]. Among the outpatient smart services, the appointment registration system is the most widely used at all levels of hospitals [51], which is also consistent with the results of the questionnaire survey. In the process of talking with the experts, it was found that the installment of the smart medical services in the hospital could not only disperse crowds during peak hours and relieve the pressure of manual service, but also reduce the frequency of manual operation errors. For the public, the correct use of smart services when seeing a doctor could speed up the entire consultation process, allow patients to better arrange their own time, and at the same time increase trust in the hospital to achieve a win-win situation.

However, with the aging of the population, the average annual number of medical consultations in Taiwan has continued to increase [6]. According to statistical analysis, the utilization rate of hospital smart services has not increased significantly [18]. Experts have pointed out that although hospital smart service systems have been popularized in large hospitals, they are not yet available in many local hospitals and clinics due to complex equipment needs as well as high costs, regulations, and policies. Moreover, the smart medical service systems in each hospital are customized by the manufacturers to make them suitable for hospitals with different operation methods and functions. At the same time, smart services still lack friendliness in their operation. In the study we also found that some elderly patients are still more accustomed to using traditional service methods, and they may ask a caregiver or hospital volunteer to help them in using smart services. Some patients come to the hospital to seek psychological treatments, and they need to communicate with doctors to relieve their inner anxiety instead of receiving medical diagnoses and treatments [52]. Medical technology is a way to help human beings improve health and reduce diseases, but the development of science and technology is a double-edged sword that challenges human spiritual life and traditional concepts for users who are not accustomed to using intelligent technology, and may even reduce the communication opportunities between people [53,54]. Therefore, smart services and humanized care should complement each other, as both are indispensable.

2. Patients’ willingness to use hospital smart service systems and the influencing factors

The research results showed that most of the respondents had used smart services for medical treatment. Among them, online appointment registration was the most frequently used, followed by self-payment kiosks. Self-service kiosks for prescription refills were special in their functionality, as the hospital set up a self-service kiosk for chronic disease patients to refill prescriptions for the needs of patients visiting regularly to get medicine. Elderly patients with chronic diseases are more accustomed to using manual services, and the customers of the self-service prescription refill kiosks were mainly family members or caregivers.

The questionnaire survey clearly showed that the variable which mainly affected the patients’ attitude toward using the hospital smart service system was their perceived usefulness; that is to say, the patients were concerned with whether the use of the smart service was simple, and they paid more attention to the benefits and convenience brought by smart services. These results were consistent with the results of previous studies in related fields [55,56,57]. Furthermore, the variable that most affected the patients’ behavioral intention was their attitude, indicating that compared with other variables, the patients trusted the use of the smart service system. This perception was beneficial to their own behaviors, as it could increase their willingness to use the smart service system.

3. Design strategies for hospital smart service systems

The potential design needs of medical services were found after the statistical analysis of the respondents’ perception and attitude, in combination with the content of the expert interviews. Patients who come to a hospital for medical treatment may show cultural differences due to coming from urban and rural areas, as well as coming from domestic and foreign areas. Using NTUH Yunlin Branch as an example, most of the patients who go there are middle-aged and elderly people. Compared with using online registration or self-payment kiosks, these patients are more accustomed to using traditional telephone registration and cash payment systems. On the other hand, in the Taipei branch of National Taiwan University Hospital, the patients who come to the hospital for treatment have a higher acceptance and experience of using smart devices compared with those coming from non-urban areas, and they are more adaptable to electronic transactions and online service methods. Therefore, regarding the cultural differences between urban and rural areas, the process of the hospital smart service system should be designed based on local differences to help local people better adapt to the smart service system. Secondly, the hospital smart service system should be designed from the perspective and thinking of the patients. From the results of the questionnaire, it was found that a smart service design that is intuitive, simple, and clear can maximize the benefit for patients and increase their usage intention. At the same time, such a design will reduce the probability of errors, improve stability, increase patients’ trust in the services and promote their willingness to use them. In addition, the explanation and promotion of the benefits of the smart service system will be strengthened, so as to attract the interest and attention of patients. In cognitive and behavioral research, it is believed that alternative experiences and group learning can help an individual achieve rapid learning effects. Due to differences in physical and cognitive status, when encouraged and assisted by peer groups, individuals’ internal rejection is reduced, and older individuals are able to learn more with their peers than when they try to learn alone [58]. Therefore, for elderly patients, the availability of a volunteer group of a similar age that can guide their use of the smart services will enhance their trust and willingness to use smart services. It can also break through the elderly patients’ thinking of being accustomed to using traditional service methods and connect them with new service concepts. Further, by increasing the humanized design and care of patients when using these services, patients will feel the convenience of the service in the process of medical treatment and experience the service care given by the hospital.

### Limitations and Recommendations

In the development and promotion of hospital smart services, in addition to the need to update technology and service systems, it is also important to improve the acceptance and willingness of the public to access the hospital smart service system. Many aspects remain that deserve more in-depth research and discussion in the research experience.

This study occurred during the COVID-19 pandemic, and it was impossible to conduct more in-depth interviews with patients. Therefore, it is recommended that researchers conduct qualitative interviews with patients in the future and conduct research investigations of specific groups, to obtain a more in-depth focus on the needs of the public and the existing pain points. Meanwhile, this study tends to be a preliminary investigation. Considering the particularity of the research site, we only use a brief theoretical model of TAM to facilitate better data collection. In the future, we hope to continue the study in depth, adding and testing other items and variables. Finally, this study mainly focused on a questionnaire survey conducted in the outpatient clinic area of NTUH Yunlin Branch. It is suggested that future researchers expand the scope of the study to explore the influence factors of hospital smart service systems for patients from urban and rural areas to better understand the differences between patients.

## 5. Conclusions

This study summarized the development status and trends of hospital smart service systems through a relevant literature review and expert interviews and analyzed the patients’ perception of using a hospital smart service system and the influencing factors on^ improving their usage intention according to the technology acceptance model.

Through the expert interviews, it was found that hospital smart service systems in Taiwan have been popularized in medium and large hospitals and are considered helpful in saving human resources, reducing the rate of manual errors, and improving the convenience of the consultation process. The experts suggested that smart services for hospitals should be considered more from the patient perspective in the future. As users and smart technology should be complementary to each other, it is also necessary to take into account humanized care while promoting smart services. After integrating the behavioral imagery of the technology acceptance model, it was found that the attitude which mainly affected the patients’ use of the smart service system in the hospital was perceived usefulness. That is, compared with the usability of smart services, the patients paid more attention to the benefits and conveniences of smart services. Moreover, the behavioral intention that most affected the patients’ use of the hospital smart service system was their attitude. Compared with the other variables, the patients had trust in the use of smart services, and this perception was beneficial to their own behavior; that is, it could increase their willingness to use smart services.

In response to the integration of smart technologies into all aspects of people’s lives today, this study discussed the perception and cognition between individuals and new technologies based on the topic of smart services in hospital outpatient clinics, and at the same time considered how to make the interaction between humans and machines friendlier. The results could serve as an important reference for the needs and improvement of hospital service systems in the future.

## Figures and Tables

**Table 1:** Statistics on number of hospital visits from 2017 to 2019 (Unit: person) [6].

	Year	2017	2018	2019
Gender				
Male		10,358,775	10,394,604	10,866,557
Female		11,032,152	11,081,465	11,449,677
Total		21,390,927	21,476,069	22,316,234

**Table 2:** Applications of medical self-service technologies [14,15].

	Type	Telephone & Interactive Voice Response	One-Line/Internet	Interactive Kiosks	Video/CD
Function					
Customer Service		Outpatient registration Medical information	Medical consultation Outpatient registration Personal information inquiry Diagnosis and treatment progress inquiry	Outpatient registration Outpatient check-in Queue up Data query	Health education videos Health consultant
Transaction		Voice prompt	Online Payment E-bank payment	Self-service payment ATM payment	Instructional video
Self-service		Voice self-service navigation	Route navigation Health management Health education propaganda Information reminder	Self-service check Self-measurement Self-service form printing	Information reminder Self-assessment Health management

**Table 3:** Introduction of the hospital outpatient smart service equipment.

Type	Equipment Introduction
Appointment and registration	A variety of registration methods are available in NTUH Yunlin Branch, including manual telephone registration, online registration, and on-site registration. Patients can use appropriate methods to make an appointment and register in advance.
Mobile service APP	NTUH launched the NTUH app in 2017. Patients can use it to make online appointments and registrations, check the number of waiting patients, and query medicines.
Self check-in kiosk	When reporting to the doctor, the patient can insert their health insurance card into the card reader of the self-check-in kiosk at the entrance of the clinic to complete the check-in process.
Self payment kiosk	At present, NTUH Yunlin Branch has two self-service payment kiosks at the Douliu Campus location. They are located near the cashier and registration counter and provide cash payment and debit card transfer functions, as well as operating instructions and step-by-step diagrams, but the parking payment function is not available.
Self-service kiosk for chronic disease patients to refill prescriptions	For patients with chronic diseases, NTUH Yunlin Branch has also launched self-service kiosks for prescription refills. When the patient’s condition is stable, the doctor will consider issuing a long-term prescription so that the patient can collect the drugs without needing additional outpatient diagnoses and treatment. These self-service kiosks make it convenient for patients to receive the medicine quickly, as they can refill their medication simply by presenting the prescription, and it also facilitates family members filling prescriptions for elderly patients who may be unable to leave the home.

**Table 4:** Backgrounds of expert subjects.

No.	Expert	Employer	Related Experience/Research Area	Years of Working in Related Areas
A	Mr. Pan	National Yunlin University of Science and Technology	Specialized areas: hospital operation, medical information, programming language, cloud computing Assistant Professor, Bachelor Program in Interdisciplinary Studies, National Yunlin University of Science and Technology Teaching experience: Changhua Hospital and Taitung Hospital of Department of Health and Welfare	20 years
B	Mr. Tsai	National Yunlin University of Science and Technology	Specialized areas: cognitive psychology, design for care and wellbeing, social design Assistant Professor, National Yunlin University of Science and Technology Assistant Professor, Department of Product and Media Design, Fo Guang University Assistant Designer, Galaxy Zero Information Technology Co., Ltd.	Over 10 years
C	Mr. Hong	NTUH Yunlin Branch	Occupational field: healthcare, outpatient pricing, and registration Supervisor, Registration and Casher Group, Medical Affairs Office, NTUH Yunlin Branch	20 years

**Table 5:** Questionnaire survey.

Perceived Usefulness	
A-Q1	I feel that using such smart services can increase the efficiency of my doctor visits.
A-Q2	I feel that using smart services like this allows me to better manage my time when I seek medical care.
A-Q3	I feel that using such smart services can make it easier for me to complete my medical appointments.
A-Q4	Overall, I find it helpful to use such smart services.
Perceived ease of use	
B-Q1	I find it easy for me to learn to use such smart services.
B-Q2	I think the interface of such smart services is easy to operate.
B-Q3	I find the use of such smart services to be clear and understandable.
B-Q4	I feel like I can easily use the functions of such smart services.
B-Q5	Overall, I find it easy and painless to use such smart services.
Attitude	
C-Q1	I find it enjoyable to use such smart services.
C-Q2	I think it is worthwhile to use such smart services when seeking medical treatment.
C-Q3	I think using such smart services is convenient for me to seek medical treatment.
C-Q4	I like using smart services like this when seeing a doctor.
C-Q5	Overall, I think such smart services are worth using.
Behavioral intention	
D-Q1	I think I will actively want to use such smart services when I seek medical treatment.
D-Q2	Compared to manual counter service, I think I will like to use such smart services.
D-Q3	I think I will recommend this type of smart service to friends and family.
D-Q4	I feel like I would love to use this type of smart service to get medical care.
D-Q5	In the future, I think I will use such smart services if there is a chance.

**Table 6:** Summary of the interview contents.

Factors Summarized	Key Points
Development status	Hospitals have gradually become patient-centered. Smart services have become popular in large hospitals, but each hospital has different functions. Each hospital has its own smart medical service system.
Future trends	Smart services will gradually be replaced by smartphones. It will be more and more streamlined and integrated into a complete process. Services will gradually become unmanned.
Social benefits	The hospital can reduce the flow of people, reduce the pressure, and reduce the points of contact between people. It reduces the frequency of manual errors. It makes the entire consultation process smoother. It increases convenience for both patients and caregivers.
Current usage	The devices are not friendly enough to operate. Elderly patients are more accustomed to using traditional service methods. It is easier for patients who often go to the hospital or have a technology foundation to use smart services. According to the attitude toward smart service, patients can be divided into three groups. Elderly patients are generally assisted by medical volunteers or caregivers.
Design strategies	Designers should think from the perspective of the elderly. Smart services should be friendlier, more stable, and easier to operate. It is necessary to break through traditional methods of use in order to connect with new service concepts. The smart technology service with a human factor design will be a positive assistance that meets the needs of patients. Humanized design should be taken into account while implementing smart services. Human and smart services should be complementary.

**Table 7:** Descriptive statistics of respondents.

	Demographics	No. of Respondents	% of Respondents
Gender	Female	130	64.4%
	Male	72	35.6%
Age	20–25	38	18.8%
	26–30	7	3.5%
	31–35	14	6.9%
	36–40	14	6.9%
	41–45	21	10.4%
	46–50	25	12.4%
	51 and above	83	41.1%
Education	Elementary School and below	1	0.5%
	Junior High School	3	1.5%
	High School	25	12.4%
	University	145	71.8%
	Master and above	28	13.9%
Marital	Married	134	72.7%
	Single	66	26.2%
	Widowed	2	0.9%
Occupation	Student	22	10.9%
	Agriculture and Fishery	0	0%
	Business and Industry	58	28.7%
	Government employees	15	7.4%
	Service Industry	50	24.8%
	Freelancer	22	10.9%
	Retirement	18	8.9%
	Other	17	8.4%

**Table 8:** *T*-test of the external variable of perceived usefulness.

	Standard Deviation (Mean)		*t*	Degrees of Freedom	Significance (Two-Tailed)	Significant or Not
	Male (N = 72)	Female (N = 130)				
Perceived Usefulness	0.723 (4.614)	0.734 (4.581)	0.315	200	0.753	Not significant

**Table 9:** *T*-test of the external variable of perceived ease of use.

	Standard Deviation (Mean)		*t*	Degrees of Freedom	Significance (Two-Tailed)	Significant or Not
	Male (N = 72)	Female (N = 130)				
Perceived Ease of Use	0.815 (4.327)	0.811 (4.270)	0.478	200	0.634	Not significant

**Table 10:** ANOVA analysis of external variable perceived usefulness.

	Item	Sum of Squares	Degrees of Freedom	Mean Square	F	Significance	Significant or Not
Age	Between groups	1.896	6	0.316	0.588	0.740	Not significant
	Within a group	104.801	195	0.537			
Educational level	Between groups	20.042	4	5.011	11.391	0.000	Significant
	Within a group	86.655	197	0.440			
Marital status	Between groups	0.514	2	0.257	0.483	0.618	Not significant
	Within a group	106.183	199	0.534			
Occupational category	Between groups	0.983	6	0.164	0.302	0.935	Not significant
	Within a group	105.714	195	0.542			

**Table 11:** ANOVA analysis of external variable perceived ease of use.

	Item	Sum of Squares	Degrees of Freedom	Mean Square	F	Significance	Significant or Not
Age	Between groups	7.633	6	1.272	1.991	0.069	Not significant
	Within a group	124.611	195	0.639			
Educational level	Between groups	24.749	4	6.187	11.339	0.000	Significant
	Within a group	107.495	197	0.546			
Marital status	Between groups	2.573	2	1.287	1.974	0.142	Not significant
	Within a group	129.671	199	0.652			
Occupational category	Between groups	5.096	6	0.849	1.303	0.258	Not significant
	Within a group	127.148	195	0.652			

**Table 12:** Correlation matrix for each dimension.

	1	2	3
1. Perceived usefulness	-		
2. Perceived ease of use	0.753 **	-	
3. Attitude	0.878 **	0.818 **	-
4. Behavioral intention	0.845 **	0.781 **	0.933 **

Significant level: ** *p* < *0*.01.

**Table 13:** Verification of each coefficient.

Model	Dependent Variable	Constant	Standard Coefficient β	T	Significance
1	Perceived usefulness	Perceived ease of use	0.753	16.182	0.000
2	Attitude	Perceived usefulness	0.878	25.954	0.000
3	Attitude	Perceived ease of use	0.818	20.139	0.000
4	Behavioral intention	Perceived usefulness	0.845	22.341	0.000
5	Behavioral intention	Attitude	0.933	36.578	0.000

**Table 14:** Analysis of the research hypothesis results.

Hypothesis	Description	Result
**H1**	**H1.** *External variables of patients’ perception of hospital smart service systems will positively affect perceived usefulness.*	Partially established
**H2**	**H2.** *External variables of patients’ perception of hospital smart service systems will positively affect perceived ease of use.*	Partially established
**H3**	**H3.** *Patients’ perceived ease of use of hospital smart service systems will positively affect perceived usefulness.*	Established
**H4**	**H4.** *Patients’ perceived usefulness of hospital smart service systems will positively affect attitude.*	Established
**H5**	**H5.** *Patients’ perceived ease of use of hospital smart service systemswill positively affect attitude.*	Established
**H6**	**H6.** *Patients’ perceived usefulness of hospital smart service systemswill positively affect behavioral intention.*	Established
**H7**	**H7.** *Patients’ attitudes toward using hospital smart service systemswill positively affect behavioral intention.*	Established

## Data Availability

The data in this study are available on request from the corresponding authors. The data are not publicly available due to [the privacy of the hospital and patients involved].

## References

[1] World Health Organization (2020). World Health Statistics 2020: Monitoring Health for the SDGs, Sustainable Development Goals.

[2] Deloitte (2021). 2021 Global Health Care Outlook.

[3] United Nations (2019). World Population Prospects 2019: Highlights.

[4] Ministry of the Interior (2018). Internal Statistics Bulletin, Week 15, 107.

[5] National Development Council Trend in Dependency Ratio. https://pop-proj.ndc.gov.tw/chart.aspx?c=11&uid=67&pid=60.

[6] Ministry of Health and Welfare (2020). 108 Universal Health Insurance Health Statistics.

[7] Healthcare Information and Management Systems Society Digital Health Transformation. https://www.himss.org/what-we-do-solutions/digital-health-transformation/achievement-list.

[8] National Health Commission of People’s Republic of China (2019). Hospital Smart Service Grading and Evaluation Standard System.

[9] Nikkei Japan Plans 10′ AI Hospitals’ to Ease Doctor Shortages. https://asia.nikkei.com/Politics/Japan-plans-10-AI-hospitals-to-ease-doctor-shortages.

[10] China Medical University Hospital The Quality of medical Care Is on Par with International Standards—China Medical University Hospital Passed the highest Level of HIMSS EMRAM Certification. https://www.cmuh.cmu.edu.tw/NewsInfo/NewsArticle?no=4675.

[11] Tsai J.-H. (2019). Lin Kou Chang Gung Memorial Hospital Becomes Taiwan’s First HIMSS EMRAM Level 7 Electronic Medical Record International Certification Organization.

[12] World Health Organization (2015). World Report on Ageing and Health.

[13] Meuter M.L., Ostrom A.L., Roundtree R.I., Bitner M.J. (2000). Self-Service Technologies: Understanding Customer Satisfaction with Technology-Based Service Encounters. J. Mark..

[14] Hsiao Y.-C. (2018). A Research on The Acceptance of Self-Service Technology Applied to Healthcare with Unified Theory of Acceptance and Use of Technology, UTAUT—A Case of Hospital Service Process.

[15] Hsieh C.-T. (2005). Implementing Self-Service Technology To Gain Competitive Advantages. Commun. IIMA.

[16] Mansurov B., Rosengren R. (2017). Self-Service Technologies: Investigation on How Self-Service Technologies Influence the Consumer S Perception of Quality.

[17] Hu Y. (2021). An improvement or a gimmick? The importance of user perceived values, previous experience, and industry context in human–robot service interaction. J. Destin. Mark. Manag..

[18] Center for Medical Quality Management (2020). Taipei Veterans General Hospital 109th Annual Outpatient Experience Survey Report.

[19] Armitage C.J., Conner M. (2001). Efficacy of the Theory of Planned Behaviour: A meta-analytic review. Br. J. Soc. Psychol..

[20] Fishman J., Yang C., Mandell D. (2021). Attitude theory and measurement in implementation science: A secondary review of empirical studies and opportunities for advancement. Implement. Sci..

[21] Sheeran P. (2002). Intention—Behavior Relations: A Conceptual and Empirical Review. Eur. Rev. Soc. Psychol..

[22] Chang S.-S., Chang P.-Y., Tsai C.-J., Chen H.-J., Tasi T.-Y. (2017). A Study on Relation of Experiences with Medical Services, the Quality of Physician-Patient Relationship and Loyalty. J. Glob. Bus. Openration Manag..

[23] Chandra S., Mohammadnezhad M., Ward P. (2018). Trust and Communication in a Doctor- Patient Relationship: A Literature Review. J. Healthc. Commun..

[24] Tsai H.-W., Huang S.-W., Hung Y.-L., Hsu Y.-S., Huang C.-C. (2021). Use of the Smart Lean Method to Conduct High-Quality Integrated Perioperative Management Prior to Hospitalization. Int. J. Environ. Res. Public Health.

[25] Ajzen I., Fishbein M. (1980). Understanding Attitudes and Predicting Social Behavior.

[26] Ko Y.-J. (2011). An Empirical Study of Consumer’s Behavioral Intention of Smart Phone Mobile Banking—Based on the Theory of Reasoned Action and Technology Acceptance Model.

[27] Davis F., Davis F. (1989). Perceived Usefulness, Perceived Ease of Use, and User Acceptance of Information Technology. MIS Q..

[28] Rahimi B., Nadri H., Lotfnezhad Afshar H., Timpka T. (2018). A Systematic Review of the Technology Acceptance Model in Health Informatics. Appl. Clin. Inform..

[29] Chimento-Díaz S., Sánchez-García P., Franco-Antonio C., Santano-Mogena E., Espino-Tato I., Cordovilla-Guardia S. (2022). Factors Associated with the Acceptance of New Technologies for Ageing in Place by People over 64 Years of Age. Int. J. Environ. Res. Public Health.

[30] Tsai C.-H. (2014). Integrating Social Capital Theory, Social Cognitive Theory, and the Technology Acceptance Model to Explore a Behavioral Model of Telehealth Systems. Int. J. Environ. Res. Public Health.

[31] Bunnell B.E., Barrera J.F., Paige S.R., Turner D., Welch B.M. (2020). Acceptability of Telemedicine Features to Promote Its Uptake in Practice: A Survey of Community Telemental Health Providers. Int. J. Environ. Res. Public Health.

[32] Peek S.T.M., Wouters E.J.M., van Hoof J., Luijkx K.G., Boeije H.R., Vrijhoef H.J.M. (2014). Factors influencing acceptance of technology for aging in place: A systematic review. Int. J. Med. Inform..

[33] Li W., Shen S., Yang J., Tang Q. (2021). Internet-Based Medical Service Use and Eudaimonic Well-Being of Urban Older Adults: A Peer Support and Technology Acceptance Model. Int. J. Environ. Res. Public Health.

[34] Nguyen M., Fujioka J., Wentlandt K., Onabajo N., Wong I., Bhatia R.S., Bhattacharyya O., Stamenova V. (2020). Using the technology acceptance model to explore health provider and administrator perceptions of the usefulness and ease of using technology in palliative care. BMC Palliat. Care.

[35] Su S.-R. (2004). A Discussion of Privacy in Hospital Outpatient Department.

[36] Mercer S.W., Reynolds W.J. (2002). Empathy and quality of care. Br. J. Gen. Pract..

[37] Arneill A., Devlin A. (2002). Perceived quality of care: The influence of the waiting room environment. J. Environ. Psychol..

[38] Lin S.-P. Hospital History, National Taiwan University Medical College Hospital, Yunlin Branch. https://www.ylh.gov.tw/?aid=60&pid=90.

[39] Chou L.-L. [NTU Hospital’s Yunlin Branch] Becomes a Model of Telemedicine in Remote Areas. https://ibmi.taiwan-healthcare.org/zh/member_news_detail.php?REFDOCID=0qqpsvmp418p4q0t.

[40] Kalayou M.H., Endehabtu B.F., Tilahun B. (2020). The Applicability of the Modified Technology Acceptance Model (TAM) on the Sustainable Adoption of eHealth Systems in Resource-Limited Settings. J. Multidiscip. Healthc..

[41] Wang E.S.-T., Chou N.P.-Y. (2014). Consumer Characteristics, Social Influence, and System Factors on Online Group-Buying Repurchasing Intention. J. Electron. Commer. Res..

[42] Lin H.C., Chang T.Y., Kuo S.H. (2018). Effects of Social Influence and System Characteristics on Traceable Agriculture Product Reuse Intention of Elderly People: Integrating Trust and Attitude Using the Technology Acceptance Model. J. Res. Educ. Sci..

[43] Abdullah F., Ward R., Ahmed E. (2016). Investigating the influence of the most commonly used external variables of TAM on students’ Perceived Ease of Use (PEOU) and Perceived Usefulness (PU) of e-portfolios. Comput. Hum. Behav..

[44] Park E.S., Park M.S. (2020). Factors of the Technology Acceptance Model for Construction IT. Appl. Sci..

[45] Chen C.-C. (2013). Statistical Analysis Using SPSS.

[46] Cook R.D., Weisberg S. (1982). Criticism and Influence Analysis in Regression. Sociol. Methodol..

[47] Venkatesh V. (2000). Determinants of Perceived Ease of Use: Integrating Control, Intrinsic Motivation, and Emotion into the Technology Acceptance Model. Inf. Syst. Res..

[48] Liao C., Chen J.-L., Yen D.C. (2007). Theory of planning behavior (TPB) and customer satisfaction in the continued use of e-service: An integrated model. Comput. Hum. Behav..

[49] Lee D., Moon J., Kim Y.J., Yi M.Y. (2015). Antecedents and consequences of mobile phone usability: Linking simplicity and interactivity to satisfaction, trust, and brand loyalty. Inf. Manag..

[50] Lu Y.-Y. (2016). A Study on the Behavioral Intention for the Public Use in the Responsive Web Design of Hospital Website.

[51] Chou Y. 2021 Smart Medical Survey: Exploring the Present and Future of Medical Intelligence in Taiwan. https://www.digitimes.com.tw/iot/article.asp?cat=158&cat1=20&cat2=70&id=0000627569_1mt6y88u5kc84al038xbu.

[52] Lake J., Turner M.S. (2017). Urgent Need for Improved Mental Health Care and a More Collaborative Model of Care. Perm. J..

[53] Cheng C.-M. (2005). Theoretical and Practical Research on Medical Technology Innovation and Humanistic Care Learning: Exploring Medical Behavior and Humanistic Care from Technology Innovation.

[54] Bradford N.K., Young J., Armfield N.R., Herbert A., Smith A.C. (2014). Home telehealth and paediatric palliative care: Clinician perceptions of what is stopping us?. BMC Palliat. Care.

[55] Rho M.J., Choi I.Y., Lee J. (2014). Predictive factors of telemedicine service acceptance and behavioral intention of physicians. Int. J. Med. Inform..

[56] Alice, Lin C.-C., Hsu S.-C., Chung C.-Y., Lin Y.-C., Huang P.-C., Chen C.-K. (2015). Combining Eldercare Technology with Interactive Arts Environment. Taiwan J. Phys. Med. Rehabil..

[57] Kamal S.A., Shafiq M., Kakria P. (2020). Investigating acceptance of telemedicine services through an extended technology acceptance model (TAM). Technol. Soc..

[58] Liang H.-W. (2010). A Study on Elderly Social Learning Behavior in Group Social Networks—A Case of Toy Clinic Shop.

